# Design and fabrication of modified bi-layer poly vinyl alcohol adhesive sealant film for preventing gastrointestinal leakage

**DOI:** 10.3389/fsurg.2022.1018590

**Published:** 2022-11-29

**Authors:** Erfan Dorkhani, Yasmin Noorafkan, Reza Akbari Asbagh, Maryam Okhovat, Asieh Heirani-Tabasi, Seyed Mohsen Ahmadi Tafti

**Affiliations:** ^1^ Research Center for Advanced Technologies in Cardiovascular Medicine, Tehran Heart Center Hospital, Tehran University of Medical Sciences, Tehran, Iran; ^2^ School of Chemical Engineering, College of Engineering, University of Tehran, Tehran, Iran; ^3^ Colorectal Surgery Research Center, Imam Khomeini Hospital Complex, Tehran University of Medical Sciences, Tehran, Iran; ^4^ Department of Surgery, Division of Colorectal Surgery, Tehran University of Medical Sciences, Tehran, Iran

**Keywords:** colon anastomosis, polyvinyl alcohol, bio-adhesive, sealant film, anastomosis leakage prevention

## Abstract

Anastomosis leakage is a common complication in gastrointestinal surgery associated with high mortality, morbidity, and cost to health care providers. According to the significant burdens of AL, several methods have been introduced to overcome this problem. Despite the crucial complications of the AL, current approaches, including glue-based adhesives and bio-based sealants, have certain disadvantages and newly gained attractions for solving this challenge. This study focused on fabricating a sealant structure based on poly (vinyl alcohol) film patterned with gelatin particles and evaluating for prevention of AL. Here, we used a 3D printed model for dry spraying gelatin particles in a random and oriented pattern on PVA films. The mechanical and adhesion properties of both types of films were assessed further, and the efficacy of the novel sealant was evaluated *in vivo*. The results revealed that the film with an oriented pattern provided better adhesive and mechanical properties, expression of anti-inflammatory cytokines, and collagen deposition. In conclusion, our novel sealant enhanced mechanical features and the healing process of gastrointestinal surgical anastomosis and can be considered a novel method for the prevention of AL.

## Graphical abstract

**Figure:**
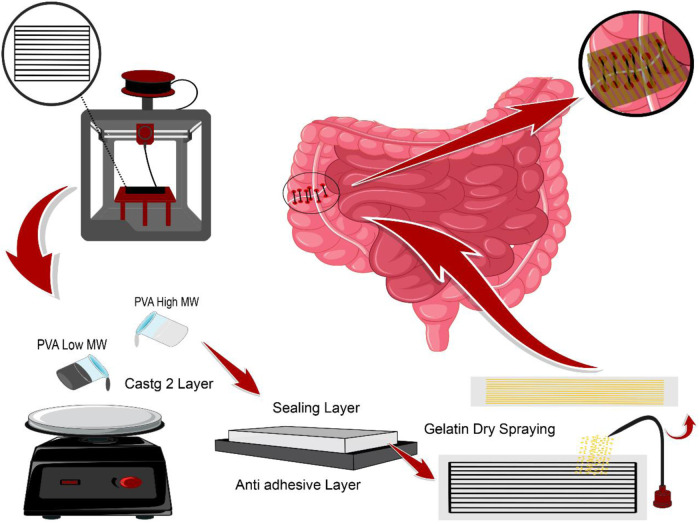
The design and fabrication of poly vinyl alcohol sealant film and evaluating efficacy of this novel patch for prevention of anastomosis leakage.

## 1 Introduction

Anastomosis leakage (AL) is any clinical or radiologic feature of surgical anastomotic dehiscence (1). AL occurs in 8.1%–11% of patients following colorectal surgeries and is associated with increased risk of morbidity and mortality, higher recurrence rate, and lower 5-year survival in patients as a severe complication of gastrointestinal surgery (2–7). AL's etiology depends on multiple factors, but the most important ones are insufficient blood supply, anastomosis under tension, infection, and inflammation (8–10). The colon and rectum are the most common sites for AL and other anastomosis complications in the gastrointestinal tract (11). Despite the improvement of medical technologies, the incidence of AL has remained stable over the last years, and the introduced techniques do not prevent AL effectively (12–14).

Due to the significant burdens of anastomosis leakage on patients and health care providers, different methods have been introduced to overcome this problem. One of the popular methods among clinicians is the application of sealants and adhesives (15–17) including fibrin glue with or without additional (18–20) cyanoacrylates adhesives (21, 22) and polyethylene glycol-based adhesives (23). Despite the popularity of bio-glues, several drawbacks, including inhibiting wound healing, decreasing anastomotic strength, limiting macrophage migration, neutrophil function, lack of long durability, high cost, and inaccessibility, have been demonstrated (24, 25). Hence, researchers have become popular among researchers designing adhesive films or hydrogels based on natural and synthetic polymers (26–28). In a study conducted by anthis et al., an adhesive sealant patch hydrogel based on poly (acrylamide -methyl acrylate -acrylic acid) with chemical stability was fabricated *via* the casting method after performing the polymerization. The results revealed adhesive energy of about 120 $J/m^{2}$ with perfect mechanical integrity maintenance and good anastomosis leakage prevention (28). Rosendorf et al. developed a double-layer nanofibrous patch based on PCL/PVA utilizing a needleless electrospinning device for anastomosis leakage prevention resulting in providing a good adhesion with no completion (23). The double-layer bioadhesive gastrointestinal film fabricated by Wu et al. also revealed ideal mechanical properties as well as desirable adhesion prospects (29).

One of the well-known polymers with good adhesion properties in tissue engineering is Polyvinyl alcohol (PVA). PVA is a biocompatible, biodegradable synthetic polymer from polyvinyl acetate hydrolysis with excellent film-forming properties. Its mechanical properties heavily hinge on the degree of hydrolysis and the final molecular weight of this polymer (30, 31). Due to its high hydrophilicity and biocompatibility with less toxicity, this polymer remains an ideal candidate for tissue engineering in biomedicine (32, 33).

Despite the several advantages of PVA, certain shortcomings require modifications to obtain appropriate features. Gelatin has a good reputation as a suitable material for improving poly (vinyl alcohol) properties because of its hydrophilic protein-based complex structure consisting of amino acid chains, bonding, and stabilizing with peptide bonds. This biopolymer has a colloidal and gel state in water, commonly obtained from the hydrolysis of natural collagen in mammals' skin, bones, and cartilage (34). High biocompatibility and non-toxicity are the criteria providing cell migration, proliferation, and differentiation, which turn this biopolymer into an ideal candidate for tissue engineering (18, 34).

Concerning the previous studies, many attempts have been carried out to develop a substrate for anastomosis leakage In the form of a composite structure, even a nanofibrous patch *via* an electrospinning process containing a wide variety of methods (35–36). Moreover, in electrospun substrates, the gecko-like adhesion prospect must take into consideration for the adhesion efficacy of the final structure (37). The significance of this challenge in gastrointestinal surgery provides new ways of developing substrates with diverse complications to improve their efficacy in this case. However, either the ease of processing or utilizing cost-effective materials having the potential of contributing to large-scale clinical applications has been taken for granted.

There are numerous feasible approaches such as dry spraying, solvent casting, and 3D printing, as well as materials including PVA and gelatin introduced to promote a high-performance device for biomedical applications. Therefore, this study investigated the fabrication of a bi-layer adhesive sealant film based on polyvinyl alcohol. Gelatin dry spraying through a 3d printed mesh on the PVA film surface was applied to enhance the final product's adhesion properties by inducing a porous surface. Both random and oriented distribution of gelatin powder on the PVA substrate was conducted to investigate the effect of adherence. The last platform's physical, structural, and biological properties have been evaluated for its tissue repair engineering application.

## 2 Materials and methods

### 2.1 Material design and characterizations

#### 2.1.1 Preparation of modified PVA tape

PVA solutions with 10% w/w concentration were prepared by dissolving two PVA with two different molecular weights (*M*_*w*_=89000–98000 and *M*_*w*_=31000–50000 with 98%–99% hydrolysis degree from sigma Aldrich 341584–363138) in the distilled water separately. The polymer solutions were stirred for 24 h to homogenize at 90 °C. Then, the final solutions were poured onto a plate for the spin coating at a rotational speed of 500 rpm.

A 3D printed patterning mesh model was designed and fabricated *via* an FDM 3D printer (model SIZAN 4, made in Iran) based on PLA filament with 80 × 40 × 3 mm dimensions. 0.4 g of gelatin powder (sigma Aldrich g 93911) was randomly and then orientally dry sprayed onto the surface of a low molecular weight wet PVA film as the first layer with the 80 × 40 mm dimensions using the 3D printed patterning mesh. The gelatin-modified films are then placed next to the cold stream, which is generated by a piezoelectric module in a separate chamber to evaporate the remaining moisture of the gelatin powder onto the film. Then the gelatin-modified films. While wet, we stick them on a high molecular weight cast PVA film as the bottom layer. The final thickness of the bi-layer PVA film is 0.45 microns.

#### 2.1.2 Experimental characterization of scaffolds

##### 2.1.2.1 Characterization of the PVA films *via* field emission scanning electron microscope (FESEM)

The morphology of the film was characterized by a Field Emission scanning electron microscope (FESEM, Hitachi model S-4160, Japan) after the films were coated with gold nanoparticles at an accelerating voltage of 20 kV.

##### 2.1.2.2 Swelling ratio

The swelling ratio of PVA, PVA with a random distribution of gelatin (PVA-RG), and PVA with an oriented distribution of gelatin (PVA-OG) were measured by immersing them in distilled water. Briefly, the films were cut into 10 × 10 mm with a 1 mm thickness. Then, the samples were frozen at −8 °C for 24 h and then placed into a freeze-dryer (Laboratory freeze dryer ALPHA 1–2 LD plus) for another 24 h at −55 °C to remove the moisture from them. In the following step, the final samples' dry weight was measured and then immersed in distilled water for 1,4, and 24 h. Afterward, the swelled films were weighed again by removing the excess water films with filter paper. The swelling ratio of the samples was calculated utilizing the following equation:

$$\text{\%}\;Swelling\;ratio=\;\frac{W_{s}-W_{d}}{W_{d}}\times 100$$

Where *W*_*s*_ and *W*_*d*_ Represent the swollen and dry weight of the films (6).

##### 2.1.2.3 In vitro degradation

The degradation rate is experimentally calculated *via* immersing the dried films in the 1 ml of 5% (v/v) fetal bovine serum (FBS; Corning) in DPBS at 37 °C for 1, 7, or 14 days. Each freeze-dried film was first weighted *W*_*f*_ then they were immersed in DPBS/FBS, and on days 1,3, and 7, the samples were lyophilized and weighed *W*_*r*_. The remaining mass of scaffolds was calculated using the equation below:

$$\mathrm{Remaining}\;\mathrm{mass}\;=\frac{W_{f}-W_{r}}{W_{f}}\times 100$$

##### 2.1.2.4 Mechanical properties

The mechanical properties of fabricated platforms have been assessed using SANTAM universal tensile testing device (Iran, SPM20). Each film with the required dimension (10 mm × 20 mm) was prepared and tested at room temperature with a 5 mm/min loading velocity. The stress-strain curve of each sample was recorded, and then stress, strain, and Young's modulus (E) were calculated. This test was performed to analyze all the films in wet conditions.

##### 2.1.2.5 BET analysis

Nitrogen adsorption-desorption isotherms were obtained by a BELSORP- mini II instrument, BEL Japan. The samples were degassed under flowing UHP grade nitrogen for 2 h at 80 °C. The samples' surface area and pore characteristics were determined based on the Brunauer-Emmett-Teller (BET) theory (38).

##### 2.1.2.6 Adhesive properties (adhesive testing)

The adhesive testing was performed using a mechanical testing machine applying PVA, PVA-OG, and PVA-RG films to ex-vivo porcine colon or stomach at 1 kPa pressure. Then, the samples were immersed and kept in the cultured media for 24 h. The tensile strength was measured in the following step based on the standard tensile test (ASTM F2258, fig. S6C). All tests were performed using a mechanical testing machine (2.5 kN load-cell, Zwick/Roel Z2.5) at a constant crosshead speed of 1 mm min−1 (7).

##### 2.1.2.7 MTT assay

The viability of cultured cells seeded onto PVA films was evaluated using MTT assay at 1, 7, and 14 days after cell seeding. The samples were incubated in a serum-free medium and MTT reagent for 3.5 h at 37 °C. The absorbance of produced formazan crystals was read at 570 nm after dissolving in dimethyl sulfoxide.

### 2.2 In vivo studies

#### 2.2.1 Study population

The ethics committee approved this study at the Tehran University of Medical Sciences. Twenty specified-pathogen-free male Wistar rats (weighing 250–280 gr) were kept in polypropylene rat cages (435*290*150 mm) with a light cycle of 14 h light and 10 h darkness and accessibility to good lab chow and water. Rats were maintained at room temperature with suitable humidity. All twenty rats were randomly divided into two case and control groups, and each group included five for day three and day seven after surgery follow-up.

#### 2.2.2 Surgical technique

Intraperitoneal administration of 30 mg/kg of Cefazolin (Daana pharma co, Tehran, Iran) was performed for all rats as a prophylactic antibiotic. Then rats underwent general anesthesia by single intraperitoneal injection of ketamine HCL 86 mg/kg (Gedeon Richter Ltd, Budapest, Hungary) and xylazine HCL 13 mg/kg (Bayer, Leverkusen, Germany). The abdomen was first shaved, then disinfected using 10% povidone-iodine. We performed laparotomy with an approximately 3 cm midline incision. The cecum part of the intestine was identified then the ascending colon lumen, 2 cm distal to the cecum, was cut sharply with a number-11 bistoury blade. For the control group, end-to-end hand-sewn anastomosis was made with 5/0 proline (Ethicon, Norderstedt, Germany). For the case group, the two edges of the cut lumen were approximate and aligned by four simple stitches, and then the whole surface of the anastomosis segment was covered tightly with the modified PVA patch. The abdomen cavity was irrigated with isotonic saline, then closed *via* a continuous suture technique with 4/0 silk (Ethicon, Norderstedt, Germany).

#### 2.2.3 Clinical outcomes

Daily examination of rats for signs of peritonitis was carried out before the second follow-up surgery. The abdomen cavity of rats was reopened on day-3 and day seven after surgery for both case and control groups (each group consists of 5 rats). After adhesion bands were cut in relaparotomy to investigate macroscopic parameters, the anastomosis was evaluated for any signs of leakage, fistula, and infection. A surgeon also observed the anastomosis site integrity. Before euthanizing rats, the Anastomosis segment was resected for histopathologic and real-time- reverse transcription-polymerase chain reaction (RT-PCR) evaluations. At the end of the surgery, each rat was sacrificed by cardiac puncture.

#### 2.2.4 Histopathologic evaluation

The specimens were preserved in a 4% formaldehyde solution. We performed Masson's trichrome staining to evaluate the collagen fibers deposition in specimens obtained from colon tissue.

To assess the TNF-a expression as a pro-inflammatory cytokine, immunohistochemistry staining was performed, and sections were provided from tissue samples of the anastomosis segment of the colon.

#### 2.2.5 Immunohistochemistry (IHC)

This study assessed the expression of tumor necrosis factor–*α* (TNF-α) using immunohistochemistry (IHC) techniques. For this purpose, specimens were collected and washed with PBS at 5 min intervals. For antigen retrieval, samples were put in 2 normal hydrochloric acid (HCL) solutions for 30 min. Afterward, Borate buffer was added to the samples to neutralize the acidic effect of HCL. Then, specimens were washed with PBS, and a 3% (v/v) Triton X-100 solution was used for 30 min to enhance cell membranes' permeability. Then, PBS was applied to the washing samples. Subsequently, goat serum 10% (v/v) was added to samples and left for 30 min to block the secondary antibody reaction. Afterward, a primary antibody diluted 1:100 in PBS was added to samples and kept at 2 °C–8 °C temperature for one day. After that, a secondary antibody diluted at 1:150 was added to the samples, and the mixture was incubated for an hour and a half at 37 °C temperature. After that, 4′,6-diamidino-2-phenylindole (DAPI) was added in a dark room, and PBS was poured over the samples promptly. Afterward, samples were divided into five areas for cell calculation by an Olympus Fluorescent microscope (×400), then images were captured from each section and analyzed using the ImageJ software (Fiji version). The percentage of positive immunolabeled cells to total cells ratio is provided as the result of these proceedings.

#### 2.2.6 Real-Time rt-PCR

In this study, RT-PCR assessment was used for the analysis of TNF-α, tumor growth factor-*β* (TGF-*β*), Interleukin-10 (IL-10), and nuclear factor kappa B (NF-*κ*B) pathway gene expression levels. This procedure contains four main phases. At first, total RNA was derived utilizing a Qiazal reagent (Qiagen, Germany). Then, one microgram of mRNA was reversed and transcribed following the manufacturer's instructions. Afterward, an Applied Biosystems 7,300 Fast Real-Time PCR System with SYBER green PCR master mix (Applied Biosystems. CA, USA) was administered for real-time RT-PCR examination. The primer sequences are reported in the supplementary material. The thermocycler's thermal cycling parameters were 95 °C for 15 min for DNA polymerase activation. After that, 45 cycles of amplification at 94 °C for 15 s, 60 °C for 15 s, and 72 °C for 30 s were performed. For the last step, melting curve analysis was carried out to verify whether all primers provided a single PCR product.

Glyceraldehyde-3-phosphate dehydrogenase (GAPDH) was used as a reference gene to normalize sequences. At last, two methods were performed to analyze relative changes in TNF-α, TGF-*β*, IL-10, and NF-*κ*B expression levels.

#### 2.2.7 Statistical analysis

The IBM ® SPSS® 26 software program was used for the statistical analysis. The results were analyzed using an independent samples *t*-test. The data are provided as means ± standard deviation (SD), and the statistical significance was *P*-value < 0.05.

## 3 Results

### 3.1 Material characterizations

#### 3.1.1 Fabrication of PVA-based adhesive tapes

#### 3.1.2 Morphological analysis

**Figure 1:**
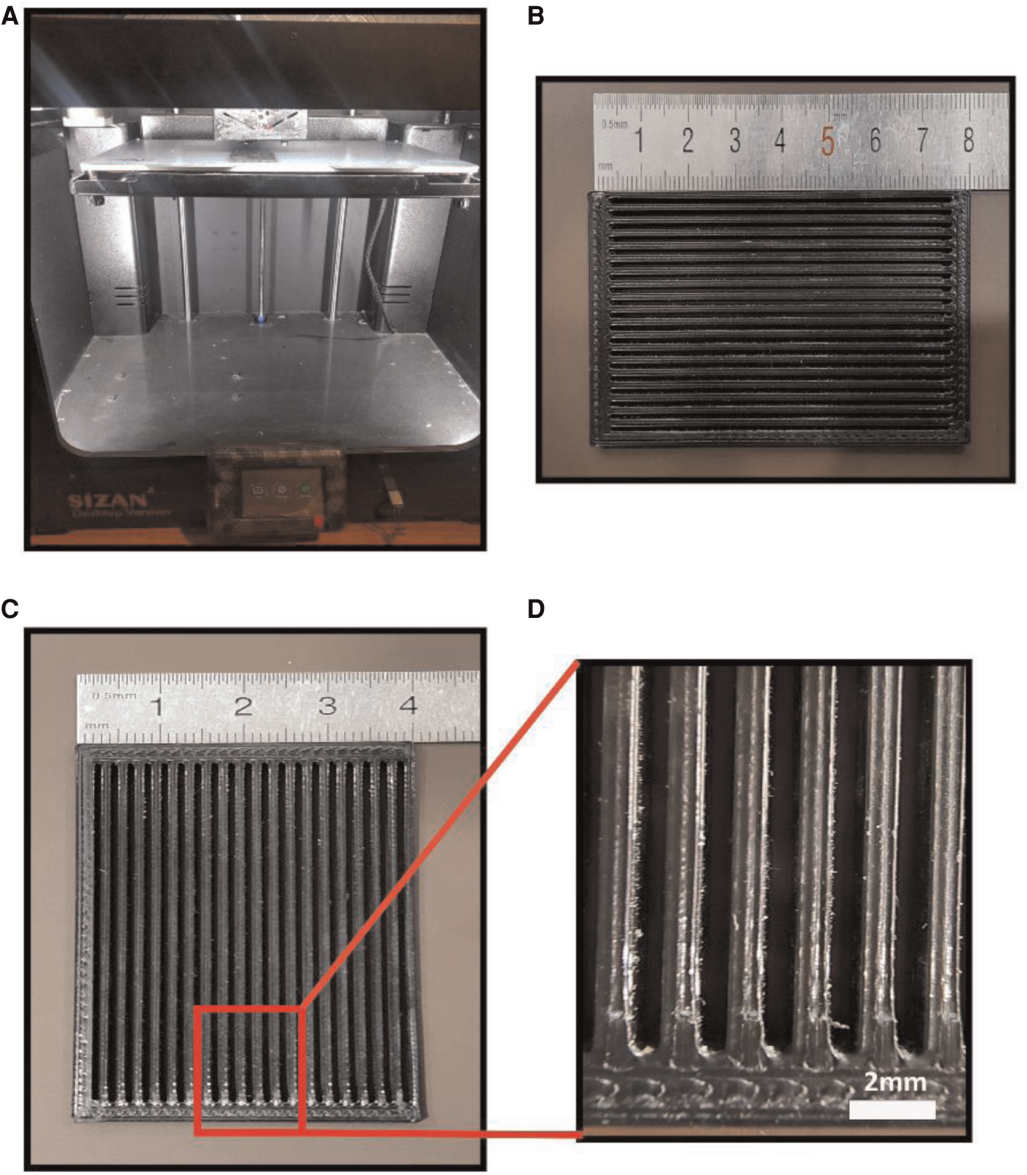
(**A**) the 3D printer utilized in this study (**B**) the 3d printed model for dry spraying gelatin particles (**C**) magnified view of 3D printed model.

**Figure 2:**
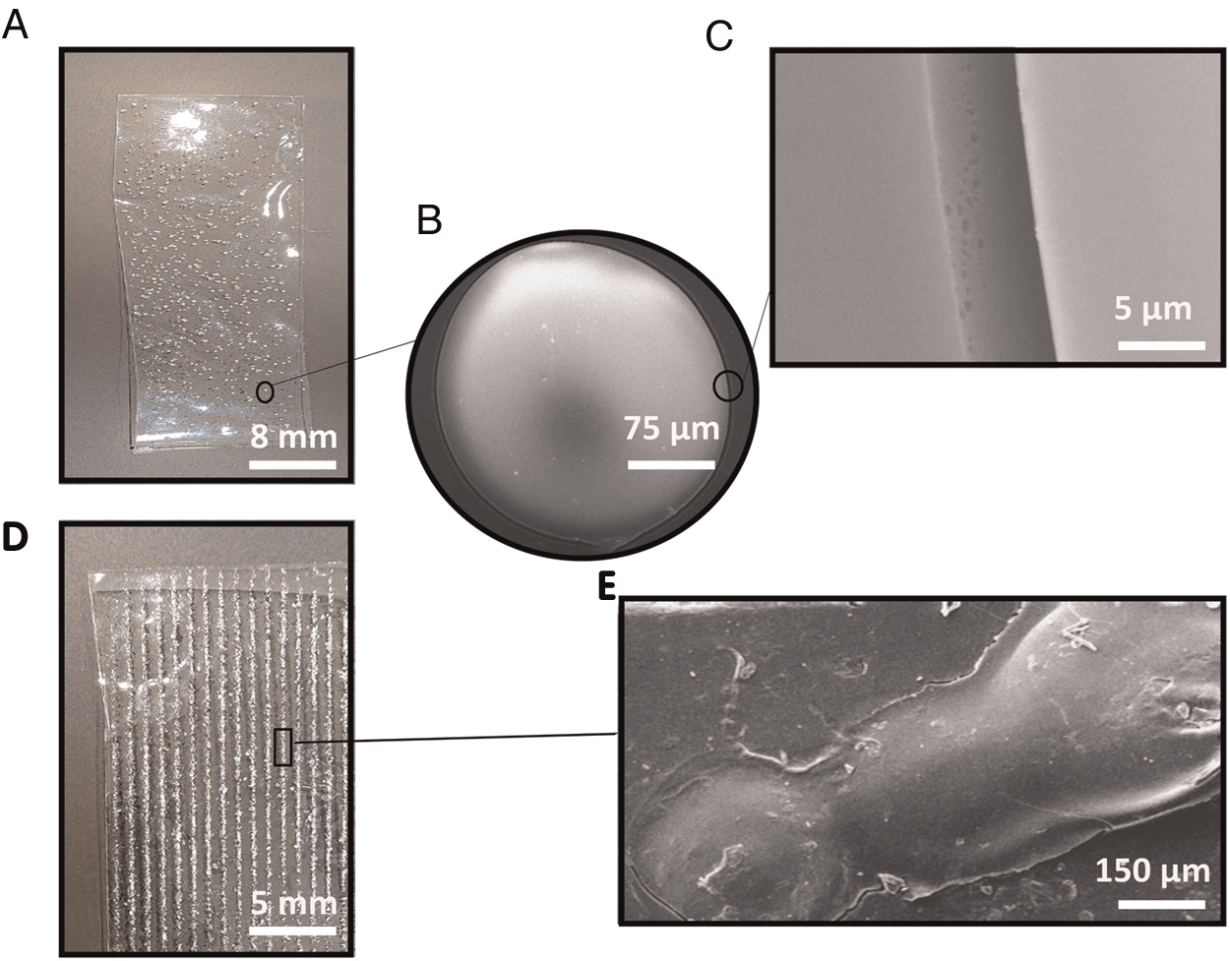
The surface characteristics of adhesive films (**A**) PVA-RG film with a random distribution of gelatin particles (**B**) the magnification of gelatin particles on the PVA-RG film (**C**) the phase separation between gelatin particles and PVA film on the PVA-RG sample (**D**) PVA-OG film with an oriented distribution of gelatin particles (**E**) the phase separation between gelatin particles and PVA film on the PVA-OG sample.

Figure 2 represents the SEM images of PVA films with different distributions of gelatin on the surface. In Figure 2B, the gelatin particle on the PVA with a random distribution of gelatin (PVA-RG) sample is detectable, potentially contributing to the formation of multiple stress concentration areas in the entire structure demonstrated in Figure 2C. Moreover, Figure 2C exhibits a non-integrated phase separation between PVA and gelatin that influences the formation of stress concentration µ areas in the final structure. Instead, in the PVA with an oriented distribution (PVA-OG) film shown in Figure 1D, a continuous and integrated phase separation can be observed in Figure 1E.

#### 3.1.3 Water absorption, *in vitro* degradability, and MTT assessment

**Figure 3:**
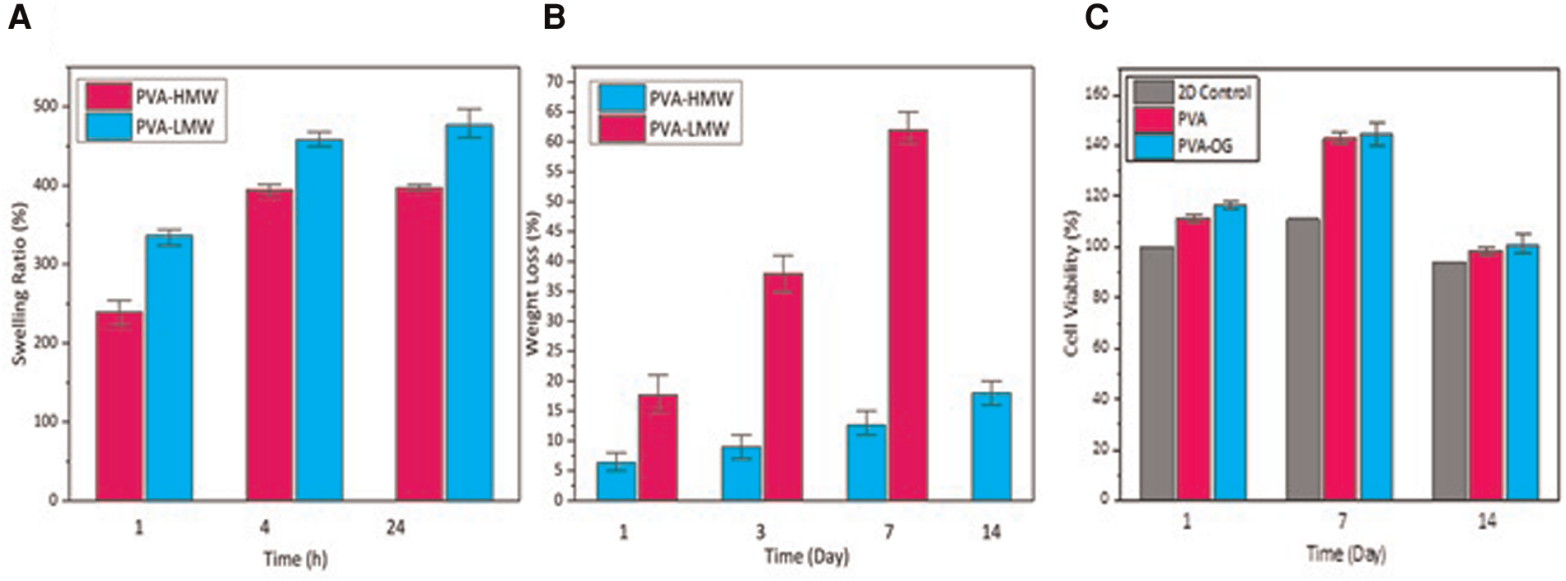
(**A**) the percentage of water absorption of PVA films with different molecular weights after being immersed in deionized water for 1,4 and 24 h (**B**) the weight loss percentage of PVA films immersed in PBS at 37 °C in days 1,3,7 and 14 (**C**) The percentage of cell viability on PVA films in 14 days.

In Figure 3, the swelling behavior and weight-loss trend of PVA films with different molecular weights (high molecular weight (HMW) and low molecular weight (LMW)) are described. Figure 3A exhibits a higher swelling ratio (about 500%) for low molecular weight PVA film after 24 h of immersing in deionized water while, in Figure 3B, we can see that PVA with low molecular weight represents a higher degradability rate during the 14 days (about 65%). This phenomenon indicates that higher molecular weight induces longer polymeric chains with more entanglement providing higher mechanical strength and dimensional sustainability (39).

Moreover, Figure 3C demonstrates the cytotoxicity of PVA and PVA-OG films during 14 days.

It can also be determined that the cell viability of cultured cells on PVA and PVA-RG films was increased until day seven while it underwent a decreasing trend on day 14 due to degradation of the low molecular weight PVA as the top layer (30).

#### 3.1.4 BET analysis

**Figure 4:**
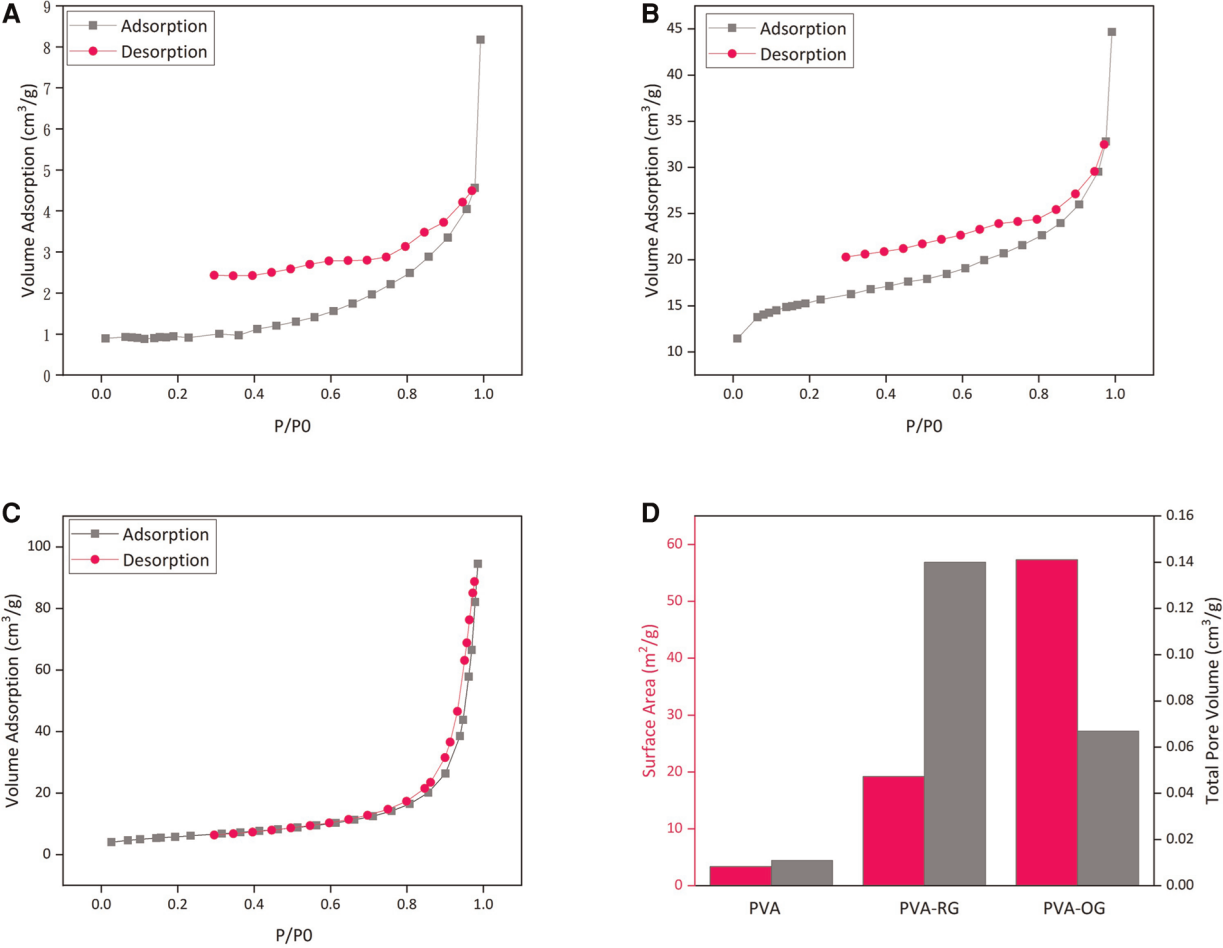
The adsorption and desorption curve of PVA films (**A**) PVA-RG, (**B**) PVA, (**C**) PVA-OG, (**D**) The changes of surface area in PVA, PVA-RG, and PVA-OG films.

Figures 4A–C) illustrates the adsorption and desorption curves for PVA-RG, PVA, and PVA-OG films. Higher adsorption and desorption volume are detectable for PVA-RG and PVA-OG samples. Moreover, Figure 4D represents the samples' total pore volume and surface area. Following these data, the adsorption and desorption curves for the gelatin samples were raised. The same trend can be observable for surface area and the total pore volume. It can be claimed that gelatin incorporation increases surface roughness and area, influentially developing surface porosity. In the PVA-RG sample, both the surface area and total pore volume have leveled up, indicating the increased surface area; however, for PVA-OG film, the oriented distribution of gelatin particles brings about the formation of double porosity due to the aligned aggregation of gelatin particles on the surface. This state leads to the depletion of pore diameters.

#### 3.1.5 Mechanical and adhesion properties

**Figure 5:**
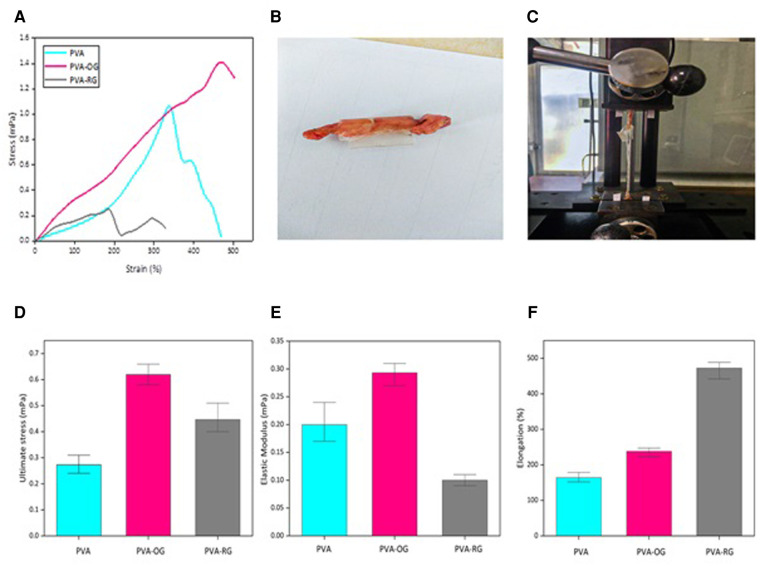
Represents both the mechanical and adhesion properties of PVA films performed *via* tensile test. The adhesive properties of PVA-based films are evaluated by performing tensile strength in the ex-vivo conditions (**A**) Stress-strain curve (**B**) The initial sample (**C**) Performing tensile test under ex vivo conditions (**D**) Ultimate stress strength (**E**) Elastic modulus (**F**) Maximum elongation.

As can be observable in Figure 5A, representing the stress-strain curve of PVA films, the PVA-OG sample reveals the highest mechanical properties with maximum stress strength of about 1.4 mPa, while the PVA-RG film shows the less (about 1.1 mPa); however, both of them have higher stress strength compared to pure PVA film. Thanks to gelatin adherence to the PVA film surface by applying vapor, a desolvation occurs, which in the PVA-RG sample leads to the formation of stress concentration. Instead, in the PVA-OG sample, the integrated and oriented distribution of gelatin powder on the PVA film surface leads to a continuous morphology, effectively dwindling the stress concentration in the final structure. Therefore, higher mechanical properties can be obtained in the PVA-OG sample, similar to the properties of fiber-reinforced polymer-based composites with unidirectional fiber orientation (40–50).

Furthermore, thanks to the random distribution of gelatin particles on the PVA-RG sample, the segregated phase separation between these two components leads to rapid fragmentation in the body fluid, inducing a faster degradation rate. However, thanks to the oriented distribution of gelatin, the PVA-OG sample developed an integrated and continuous structure in the entire sample, increasing the deformation resistance (42, 43, 45, 46, 48–50).

Additionally, Figures 5B–F reveals the adhesion property of PVA-based films. The results exhibit that PVA-OG film has an ultimate stress of about 0.6 mPa, modulus close to 0.3 mPa, and ultimate stress of around 0.6 mPa exhibiting the highest adhesion property due to the aligned distribution of gelatin. As discussed before, the incorporation of gelatin particles in an oriented distribution leads to lowering the stress-concentration areas in the entire structure, affecting the enlarged surface area and the increased porosity. This statement increases surface roughness, resulting in adhering to the native tissue.

### 3.2 In vivo experiments results

#### 3.2.1 Macroscopic results

**Figure 6:**
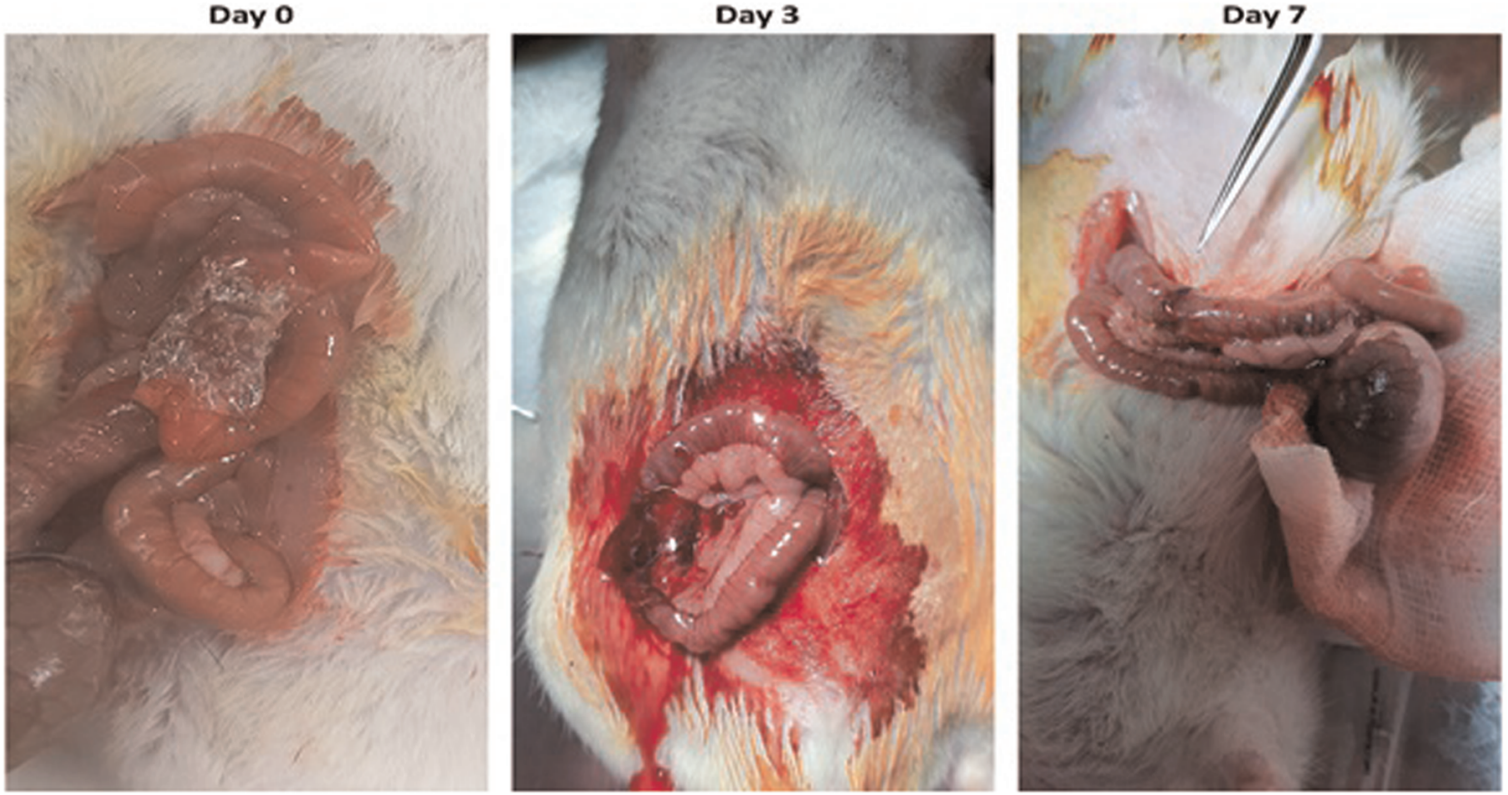
The PVA sealant implemented at the site of anastomosis on day 0. The evidence of PVA biodegradation is seen on days 3 and 7.

All animals survived with an ideal clinical situation and no complications during the follow-up. The feeding quality was good in both groups, similar to each other. There was no ileus, sepsis, or other immunological reactions to the synthesized patch. All the animals had fecal passage. This macroscopic data revealed that the patch was safe without any adverse outcomes. Additionally, the PVA sealant degraded successfully after seven days of follow-up (Figure 6).

#### 3.2.2 Microscopic pathologic images (tc, IHC)

**Figure 7:**
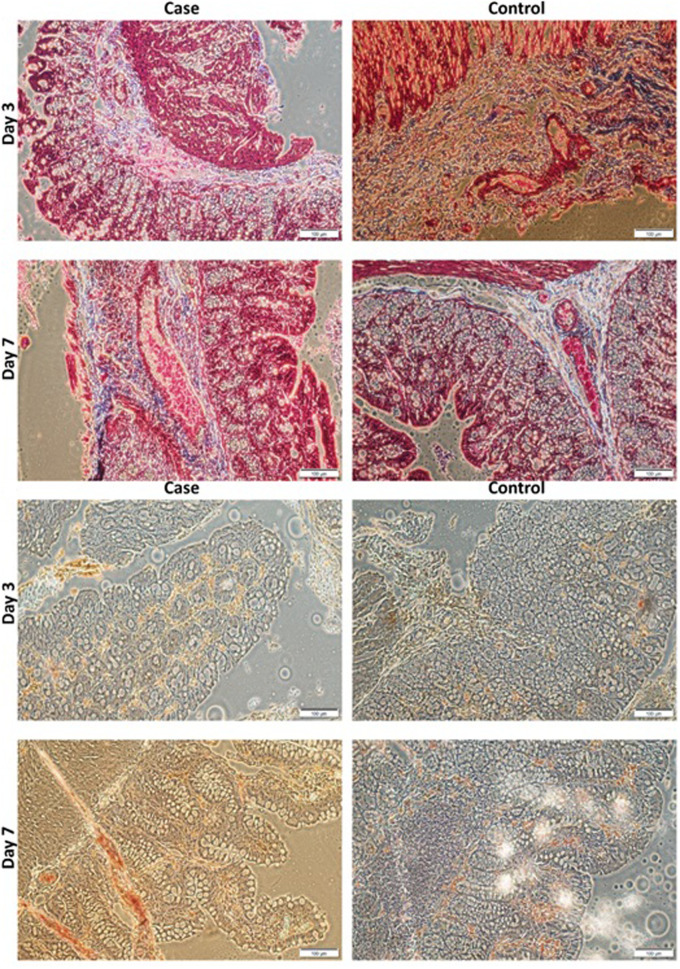
(**A**). Masson's trichrome staining of PVA and control groups at day 3 and 7 POD (X 40). (**B**). Immunohistochemistry staining for expression of TNF-α of PVA and control groups at days 3 and 7 POD (X40). Scale bar:100 µm and 0.1 mm. PVA: Polyvinyl Alcohol.

The result of immunohistochemistry staining for evaluating TNF-α expression (Figure 7, 8 a, and b) illustrated that the expression of TNF-α in the scaffold group was significantly lower than in the control group at the days three post-operation (*p*-value 0.007). However, there was no statistically significant difference on day 7. In addition, Masson Trichrome staining revealed that scaffolds lead to statistically significant higher collagen deposition than the control group on days 3 and 7 (*p*-value = 0.004 and 0.001, respectively)**.**

#### 3.2.3 RT-qPCR

To assess the role of the scaffold in the suppression of inflammation, we performed RT-qPCR to evaluate the expression of inflammatory and anti-inflammatory agents. The evaluations revealed that the expression of TNF-α in the scaffold group was the same as in the control group on day seven post-operation. However, the scaffold group demonstrated significantly lower levels of TNF-α expression than the control group on day three post-operation (*p*-value < 0.001). In addition to TNF-α, we measured the expression of the NF-*κ*B transcription factor as an immune-activator factor. The scaffold group expressed NF-*κ*B factor lesser than the control the group at days 3 (*p*-value < 0.001) and 7 (*p*-value = 0.009) post-operation. In addition to inflammatory cytokines, we assessed the expression of anti-inflammatory cytokines, including TGF-*β* and IL-10. The evaluations demonstrated that the expression of IL-10 and TGF-*β* on day three post-operation was similar between the control and staff in the old groups. At the same time, the expression of IL-10 and TGF-*β* as anti-inflammatory cytokines in the scaffold group was significantly higher than in the control group on day 7 post-operation (*p*-value < 0.001).

## 4 Discussion

### 4.1 Material design and characterization

We could successfully develop an adhesive sealant film based on PVA patterned by gelatin powder providing proper support for anastomosis leakage. Before this study, numerous types of research have carried out to find the best remedy for this issue, most of them focused on applying bio-glues. Although many studies proved the bio-glues practical potential for AL, there are indisputable disadvantageous among these materials, including limited potential for providing long durability, expensive processing, and unattainability. Therefore, we are motivated to fabricate a sealant film with a novel design utilizing dry spraying to increase the efficacy of the sealing property. Dry spraying provides the polymeric film surface with high porosity improving the adhesion property. This circumstance enables the enhancement of fluid absorption such as blood or body fluids. The results demonstrated that the oriented distribution of gelatin particles on PVA film (PVA-OG) contributes to an integrated continuous phase separation between these polymers. Moreover, the highest mechanical property, as well as conventional adhesion properties, were achieved from this sample. To put our achievement under precise evaluation, we listed some related previous studies to provide a basis for comparison.

Anthis et al. conducted a previous study on fabricating a chemically stable, adhesive sealant hydrogel patch for intestinal anastomosis leakage prevention based on acrylate compositions. Radical polymerization of acrylamide, methyl acrylate, acrylic acid, and bis-acrylamide using a low dose of UV light was applied to fabricate the intended hydrogel. The results revealed a compact structure with 59% porosity whose swelling ratio increased by 27 units after 24 h of being immersed in PBS. The dynamic modulus of hydrogels in the in-situ condition was evaluated at around 300 pa while exhibiting a viscoelastic behavior with a fracture-proof structure. The *in vivo* studies also confirmed the biocompatibility of the final products (28).

In another study conducted by Mizuno et al. through the Fabrication of modified Alaska Pollock-derived gelatin to obtain a tissue sealant hydrogel with the anti-adhesion property. The results confirmed the viscoelastic behavior of hydrogels whose elastic modulus was 50 kPa with a damping fabioadhesive 0.032. The swelling ratio was achieved at about 20% after 24 h of immersion in PBS. The ex vivo anti-adhesion evaluation also exhibited a proper bonding strength of around 2 kPa after 180 s (18).

Additionally, Wu et al. studied the fabrication of a ready-to-wear two-layer bioadhesive patch for sutures restoration. The top layer consists of a hydrophilic polyurethane to induce non-adhesion criteria. At the same time, covalently crosslinked poly acrylic acid-(NHS) was deployed to develop a dry bioadhesive property for the bottom layer, whose mechanical properties were reinforced *via* a physically crosslinked PVA layer. The shear and tensile strength were calculated at 80 and 65 kPa, respectively (29).

Compared with the previous results, thanks to the novel design of films inspired by the fiber-reinforced polymeric composites, our findings indicate much higher mechanical properties with better adhesion properties enabling conventional sealing attributes. It has been proved that PVA is synthesized from the hydrolysis of poly (vinyl acetate), contributing to the formation of hydroxyl groups in its primary structure. This phenomenon plays a determinative role in developing adhesion criteria in the material. In other words, the more hydroxyl groups, the more adhesion property. Similarly, gelatin is well-known as a protein-based adhesive thanks to its amine groups. Thus, the incorporation of gelatin particles in the PVA matrix by oriental patterning has a lot to promote both mechanical and adhesion properties of the final platform. Therefore, PVA achieved either higher mechanical or adhesion properties.

It can be claimed that this project potentially implements a facile promising strategy for fabricating sealants with higher efficacy.

### 4.2 In vivo evaluations

In this study, we successfully created a model of the anastomosis with a defect in the ascending colon of rats. The defect was small enough to cause a technical fault as a factor for AL and large enough to impair the healing process (15). The location of the anastomosis was chosen based on its suitable accessibility and avoiding complications not related to it, such as minor abdominal wounds. As the AL is an early complication usually occurring within the first ten post-operation days (16), a 7-days follow-up period was considered to evaluate histological and macroscopic outcomes of the intervention. However, the long-term assessment of this novel patch regarding its biodegradability and late complications needs longer observation times.

Due to the significant burdens of anastomosis leakage on patients and health care providers, before this study, different methods were introduced to overcome this problem. One of the clinicians' popular methods is applying fibrin glue (FG) or fibrin sealant. The FG promotes anastomosis healing by reinforcement of clot formation. The previous studies have demonstrated that FG has positive effects on anastomosis healing, but its efficacy in preventing AL is unclear (17). Additionally, further investigations have shown that the FG did not significantly influence anastomosis healing and its effects on healing are mainly based on its mechanical sealant (19). To evaluate the efficacy of a novel tissue-sealant, Mizuno et al*.* introduced a novel sealant composed of ecyl group-modified gelatin (C10-ApGltn) and a poly (ethylene glycol)-based crosslinker. C10-ApGltn-based hydrogel (C10-gel). This novel sealant demonstrated the prevention of cell infiltration at the injury site. However, it is not assessed whether the sealant reduced the AL *in vivo* or not (18). Another method for AL prevention is applying nanofibrous materials, mainly because of their effects on wound healing (21). Rosendorf et al*.* demonstrated that applying a double-layered polycaprolactone and polyvinyl alcohol nanofibrous promotes anastomosis healing in animal models. Similar to our study, there was no AL in both control and case groups, and because of that, they could not assess the role of double-layered nanofibrous in preventing AL (23).

Growth factors and cytokines highly regulate the production and releasing of MMPs and other components of anastomosis healing. To investigate the underlying mechanism for the healing effects of our novel patch on colon anastomosis, immunohistochemistry, and RT-PCR analyses were performed in the present study. Therefore, we measured levels of different cytokines contributing to AL. TNF-α is an inflammatory cytokine released by macrophages and *T*-cells in response to tissue injuries and infections (24). Fuda et al. and Yamamoto et al. demonstrated that the rise in TNF-α levels on day three post-operation is associated with a higher risk of anastomosis leakage (25, 26). Our study demonstrated that the TNF-α levels, as a predictor cytokine for AL, were lower in the experimental group in the IHC staining and RT-PCR results.

Additionally, the expression of TNF-α was lower in the experimental group on day three post-operation. It can be concluded that the application of the novel patch leads to lower levels of TNF-α cytokine as a predictor factor for AL. Transforming growth factor *β* (TGF-*β*) is an anti-inflammatory cytokine released from different cells. The TGF-*β* is beneficial in intestinal anastomosis by improving migration, proliferation, ECM synthesis, and inhibition of ECM degradation by regulating MMP activity (51). In our study, the patch application leads to the expression of TGF-*β* more than in the control groups, especially on day seven post-operation. This finding suggests that the patch improves ECM synthesis, which is compatible with the results of MT staining. Interleukin 10 (IL-10) is an anti-inflammatory cytokine that can reinforce anastomosis healing by suppressing the expression of inflammatory cytokines (20). Activating the NF- *Κ*B signaling pathway leads to the recruitment of immune cells and inhibits anastomosis healing (10). As demonstrated in our results, our novel patch downregulates the activation of the NF- *Κ*B signaling pathway.

The evaluations of our study had certain limitations. The first limitation was the absence of peritonitis and AL complications in both control and case groups. Because of this limitation, we can not conclude that our patch reduces the possibility of AL complications. Another limitation was related to the amount of the study population. The larger study population can better assess the efficacy of our novel patch for the prevention of AL.

## Data Availability

The original contributions presented in the study are included in the article/**Supplementary materials**, further inquiries can be directed to the corresponding author/s.
