# Supplementary material for: Design and fabrication of modified bi-layer poly vinyl alcohol adhesive sealant film for preventing gastrointestinal leakage
Source: Front Surg. 2022 Nov 29;9:1018590. doi: 10.3389/fsurg.2022.1018590 (PMC9744773; doi:10.3389/fsurg.2022.1018590)
Supplement: Supplementary file 1 [file Table1.docx]

| Gene name | Primers (Forward and reverse) |  |
| --- | --- | --- |
| GAPDH | GATGGTGATGGGTTTCCCGT | 262 |
|  | GCATCTTCTTGTGCAGTGCC |  |
| TNFα | GAAAGCATGATCCGAGATGT | 108 |
|  | CAGGAATGAGAAGAGGCTGA |  |
| NF-KB | TTCCCTGAAGTGGAGCTAGGA | 185 |
|  | CATGTCGAGGAAGACACTGGA |  |
| TGF-β1 | GCTAATGGTGGACCGCAACAAC | 100 |
|  | CACTGCTTCCCGAATGTCTGAC |  |
| TLR4 | GACCTCAGCTTCAATGGTGTC | 199 |
|  | TCAAGCCAAGAAATATGCCATC |  |
| STAT3 | CGCCTTGGATTGAGAGCCAAGAT | 112 |
|  | AGGAATCGGCTATACTGCTGGT |  |
| IL-10 | GCAGACAAACAATACGC | 264 |
|  | ACTTGCCCTCATCCC |  |
| SOD | AGCTGCACCACAGCAAGCAC | 191 |
|  | TCCACCACCCTTAGGGCTCA |  |
| NRF2 | ATCCTTTGGAGGCAAGACAT | 137 |
|  | TCCTGTTCCTTCTGGAGTTG |  |
